# Prediction of the Human EP1 Receptor Binding Site by Homology Modeling and Molecular Dynamics Simulation

**DOI:** 10.3797/scipharm.1106-24

**Published:** 2011-08-07

**Authors:** Behnoush Zare, Armin Madadkar-Sobhani, Siavoush Dastmalchi, Masoud Mahmoudian

**Affiliations:** 1 Department of Pharmacology, Faculty of Medicine, Tehran University of Medical Sciences, Tehran, Iran; 2 Department of Bioinformatics, Institute of Biochemistry and Biophysics, University of Tehran, Tehran, Iran; 3 School of Pharmacy and Biotechnology Research Center, Tabriz University of Medical Sciences, Tabriz, Iran; 4 Department of Pharmacology, Firoozgar Hospital, Clinical Research Development Center, Tehran, Iran

**Keywords:** Homology modeling, G-protein coupled receptors (GPCR), Human prostanoid E1 receptor, Flexible-ligand docking, Molecular dynamics simulation

## Abstract

The prostanoid receptor EP1 is a G-protein-coupled receptor (GPCR) known to be involved in a variety of pathological disorders such as pain, fever and inflammation. These receptors are important drug targets, but design of subtype specific agonists and antagonists has been partially hampered by the absence of three-dimensional structures for these receptors. To understand the molecular interactions of the PGE2, an endogen ligand, with the EP1 receptor, a homology model of the human EP1 receptor (hEP1R) with all connecting loops was constructed from the 2.6 Å resolution crystal structure (PDB code: 1L9H) of bovine rhodopsin. The initial model generated by MODELLER was subjected to molecular dynamics simulation to assess quality of the model. Also, a step by step ligand-supported model refinement was performed, including initial docking of PGE2 and iloprost in the putative binding site, followed by several rounds of energy minimizations and molecular dynamics simulations. Docking studies were performed for PGE2 and some other related compounds in the active site of the final hEP1 receptor model. The docking enabled us to identify key molecular interactions supported by the mutagenesis data. Also, the correlation of r^2^=0.81 was observed between the Ki values and the docking scores of 15 prostanoid compounds. The results obtained in this study may provide new insights toward understanding the active site conformation of the hEP1 receptor and can be used for the structure-based design of novel specific ligands.

## Introduction

The prostanoids are the cyclooxygenase metabolites of arachidonic acid and include prostaglandin (PG) D_2_, PGE_2_, PGF_2α_, PGI_2_, and thromboxane A_2_. They are synthesized and excreted from the stimulated cells and play many important roles in a variety of physiological and pathophysiological processes in the body upon interacting with their specific receptors on the effector cells [1]. The prostanoid receptors consist of eight types, each encoded by different genes and the analyses of their primary structures demonstrate the presence of seven hydrophobic transmembrane (TM) regions which is common structural architecture for G-protein-coupled receptors (GPCRs) [2]. The existence of multiple PGE_2_ receptor subtypes, namely EP1, EP2, EP3 and EP4, has been confirmed by molecular cloning [3]. Each of these receptor subtypes encoded by different genes and is probably responsible for distinct effects of PGE_2_ [4]. The human EP1 receptor (hEP1R) was originally described as a smooth muscle constrictor, involved in pain sensitization, fever and inflammation [5]. There are selective agonists that bind to the hEP1R; however, they also show significant affinity to the other receptor subtypes. Therefore, the knowledge of the structural features of EP1R is essential for the understanding of its function and for its use in drug design.

Structurally, a GPCR is characterized by the presence of a helical bundle consisting of seven hydrophobic transmembrane helices (TM1-TM7), which are connected through six alternating extracellular and intracellular loops [6]. The N-terminus is located on the extracellular side of the membrane, whereas the C-terminus occupies the intracellular side. The transmembrane domain is constituted by seven α-helices, which are known to adopt a common folding pattern, and thus, the 7TM domain represents the most conserved region among the GPCR family of proteins [7]. Several highly conserved functional microdomains and disulfide bridges are identified in the TM helices of the class A GPCRs. Some of these conserved regions include: the disulfide bond linking TM3 and extracellular loop 2 (EL2), the “Gx(3)N” motif in TM1, the “Lx(3)D” motif in TM2, the “D/ERY” motif in TM3 and the “NPx(2)Y” motif in TM7 (Table 1). These regions are known to have an important role in the structural and functional integrity of the class A (or rhodopsin-like) GPCRs to which hEP1R belongs [8]. There are some crystal structures of members of GPCRs receptors available: Bovine rhodopsin (PDB code: 1L9H) [9], β_2_-adrenergic receptor (PDB code: 2RH1**)** [10], active β_2_-adrenergic receptor (PDB code: 3P0G) [11] and CXCR4 chemokine receptor (PDB code: 3ODU) [12]. Rhodopsin is one of the GPCRs for which several three-dimensional (3D) structures have been determined by X-ray crystallography, and the coordinates are available through Protein Data Bank (PDB) [13]. Since the first high resolution structure of bovine rhodopsin became available in 2000, it has been frequently used as the template for the modeling of GPCRs [14].

Due to the lack of structural information, homology modeling has proven to be a valid alternative for constructing sound 3D models of proteins [15]. Although homology modeling was extensively performed in the case of GPCRs, the technique faces several limitations for these receptors, including poor sequence identity between target and template sequences, as well as availability of only some structural templates, namely bovine rhodopsin receptor [9], human β_2_-adrenoreceptor [10], turkey β_1_-adrenoceptor [16] and human A2A adenosine receptor [17]. This emphasizes the need for incorporating available experimental information such as site-directed mutagenesis and structure-activity relationship (SAR) data during the process of homology modeling and molecular dynamics refinement for developing an acceptable 3D model of a GPCR. A recent study demonstrated that this so-called dynamic homology modeling approach can reproduce structural and dynamic properties of the β_2_-adrenoreceptor, starting from the rhodopsin crystal structure as a template [18] and can be used to create reasonable models for the understanding of structure and dynamics of other rhodopsin-like GPCRs.

In a previous study we constructed the hEP1R model using the hβ2-adrenoreceptor as template [19]. The model had a reasonable conformation and active site properties.

In this study the structural model of full-length hEP1R was constructed via homology modeling using the crystal structure of bovine rhodopsin as the template because it will certainly keep serving the GPCR community as a prototypical receptor and a generator of ideas to be tested on other systems [20]. Also we used more methodologies based on hydrophylic and hydrophobic characteristics of membrane proteins for evaluation of the constructed models. From the three different sets of alignments, the stability of the best model was examined by molecular dynamics simulation. As a refinement process the pocket expansion method was applied to the constructed model by inserting a ligand inside the binding pocket in a position guided by the available mutagenesis data, during an extended simulated annealing MD simulation. Finally, in order to investigate binding modes, PGE_2_ and 15 other prostanoid hEP1R analogs with known binding affinity were docked into the final refined receptor model.

## Methodology

### Molecular structures

The human prostaglandin E2 receptor EP1 subtype (hEP1R, 402 amino acids) sequence was obtained from SWISS-PROT database (accession number P34995) [21]. Bovine rhodopsin crystal structure solved at 2.60 Å resolution (PDB code: 1L9H) was downloaded from Protein Data Bank (http://www.rcsb.org) and used as template for comparative modeling of hEP1R.

### TM helix prediction

TM helices within bovine rhodopsin structure were assigned using DSSP [22]. The following helix prediction methods were used directly from their websites to assign putative TM helix segments of hEP1R: HMMTOP [23], MEMSAT 1.5 [24], PHD [25], TMHMM [26], TopPred II [27], PROF [28], JPRED [29], SAM-T98 [30], PORTER [31], PSIPRED [32], SABLE [33], SCRATCH [34]. A consensus prediction for each residue was calculated by counting the number of methods that predicted the residue as being in a helix. For example consensus 100% was assigned to a residue in the protein which was predicted as helix by all of the 12 methods. The same procedure was used to predict TM helices of bovine rhodopsin for comparing with the result of DSSP.

### Multiple sequence alignment

Sequence alignments were performed using three distinct methods. First, the TM segments of the hEP1R and those of bovine rhodopsin as indicated in the GPCR database (http://www.gpcr.org/7tm) were aligned [35]. For the extra-transmembrane regions, ClustalW 1.83 [36] was utilized using a gap penalty of 10 and employing the BLOSUM62 weight matrix. The obtained alignment was called ALIGN-I. Multiple sequence alignment between bovine rhodopsin and the 41 prostanoid receptors from all species was called ALIGN-II. The third alignment (ALIGN-III) was produced via manually modifying, by SEAVIEW [37], the sequence alignment of rhodopsin receptor and hEP1R using ClustalW. These three alignments are illustrated in Fig. 3.

### Homology modeling

MODELLER 9v2 [38] was used to build homology models of hEP1R from the bovine rhodopsin crystallographic structure in the inactive state (PDB code: 1L9H), based on the three different sequence alignments described above. From the alignments, 3D models containing all nonhydrogen atoms were obtained automatically using the method implemented in MODELLER. Of the 10000 models generated with MODELLER for each alignment, the one corresponding to the lowest value of the probability density function (pdf) and fewest restraints violations was used for further analysis and named as MODELI, II and III depending on the type of alignment (ALIGN-I, II and III), respectively. An *ab initio* method implemented in MODELLER that has been demonstrated to predict the conformations of loop regions was used to build some loops of the best model. Stereochemical quality assessment of the models and the generation of the Ramachandran plot were carried out using PROCHECK [39]. The root mean square deviations (RMSDs) of the models relative to 1L9H were calculated using MODELLER. The lipid compatibility scores for helix bundles and for entire models were calculated using REPIMPS method [40]. This method, which is an adaptation of the Profiles-3D program [41, 42], an inverse-folding methodology, takes into account the fact that exposed areas of side chains for many residues in integral membrane proteins (IMPs) are in contact with lipid and not the aqueous phase. REPIMPS was tested [40] and showed useful for determining structural properties of IMPs, particularly for GPCRs [14].

Based on the π lipid propensity scale, rotational orientation angles (α), which determine the sidedness of the helices along the axis of the helical bundle, for the TM helices of the three model structures of hEP1R were predicted using the HTMSRAP method [43]. Then the percentages of the accessible surface area of the helices exposed to the lipid bilayer were measured.

### Molecular dynamics simulations

All Molecular Dynamics (MD) simulations were carried out with the GROMACS 3.3 package [44 using the ffgmx force field (GMX force field of Gromos87) at constant temperature (300 K), pressure (1 bar) and number of particles [45]. Solvent (i.e. water and ions), lipid and protein were coupled separately to a temperature bath, with a coupling constant of T=0.1 picosecond (ps). The previously equilibrated lipid bilayer by Tieleman et al. was used (available from http://moose.bio.ucalgary.ca) [46]. The best model from homology modeling (MODEL-III) was inserted at the center of the POPE (palmitoyl-oleoylphosphatidylethanolamine) bilayer with its long axis normal to the membrane-water interface. The α-helical domain of the receptor was placed at the same level as the lipid bilayer, and the eighth short helix was placed at the polar interface of the membrane. Overlapping lipid and water molecules were discarded to avoid strong repulsive van der Waals interactions. Thirty chloride ions were added to the solution in order to ensure neutrality of the entire system that comprised the hEP1R, 250 POPEs, and 14217 water molecules (a total of 59695 atoms). In this simulation His88 was in positively charged form (protonated state at NE2). Periodic boundary conditions were applied in all three directions of space. Energy minimizations were performed using a steepest descent algorithm.

Membrane equilibration was performed for 1 ns. With all protein atoms restrained, membrane and water molecules were given sufficient time to adapt to the inserted protein as described previously [47]. Temperature and pressure of the system were controlled by coupling them to the reference values of 300 K and 1 bar using time constants of 0.1 and 1.0 ps respectively. Then, the restraints were removed stepwise with two times 100 ps MD simulation. Finally a 10 ns molecular dynamics production phase was carried out on the entire system. The run parameters were the same as above.

### Ligand-supported model refinement with PGE_2_ and iloprost

A vacuum minimal energy structure of PGE_2_ prepared as described in the docking procedure section below was inserted into the selected cavity of MODEL-III as proposed by the known SAR information reported previously [18, 48]. PRODRG server [49] was used to assign atomic charges of PGE_2_ and generating the necessary topology file for GROMACS. The receptor-PGE_2_ complex was first minimized to relieve bad contacts between the non-bonded atoms. The minimization protocol for all calculations was an initial phase involving 1000 steps of steepest descent, followed by conjugate gradient algorithm, up to a gradient of 100 kJ/mol^−1^nm^−1^. The entire protein was relaxed during these simulations, except for a mild positional restraint of 10.0 kJ/Å/mol^−2^ on the backbone atoms, in order to preserve the 3D fold of the TM helices. A simulated annealing protocol was used, where the complex was “heated” gradually from a temperature of 100 to 600 K, in steps of 100 K, with 10 ps simulation at each stage. At 600 K, the complex was simulated for another 50 ps, followed by a gradual “cooling” to 300 K, in steps of 100 K, with 10 ps of simulation at each stage. Finally, a production phase was carried out involving a 50 ps simulation using an NVT ensemble at 300 K [50]. The complex was minimized as mentioned earlier. This helped to significantly open the binding site of the hEP1R model (MODEL-IV). Additional refinement was performed to achieve a more expanded binding site using iloprost, an EP1R agonist, by running the same protocol as mentioned for PGE_2_. An average structure was calculated from the final 20 ps of the production phase of the MD simulation and after being subjected to the minimization protocol as described above, the obtained model was named MODEL-V.

### Docking procedure

Three-dimensional structures of PGE_2_ and 15 other prostanoid analogues, including all hydrogen atoms, were constructed and optimized in vacuum using Polak-Ribiere conjugate gradient algorithm and AMBER95 force field implemented in HyperChem (HyperCube Inc., Gainesville, FL). Docking calculations with GOLD (Genetic Optimization of Ligand Docking) version 3.0.1 were performed using default parameters. The binding site was defined based on the known SAR data and site-directed mutagenesis information. All amino acid residues within 10 Å from the center constituted the binding site. Visual inspection was done to confirm that all important amino acids were included in the defined binding site. Each molecule was docked 100 times, and the top-ranked pose was retained for further analysis.

### Molecular images and animations

All the molecular images and animations were produced using VMD [51] and rendered by Tachyon ray tracer. Schematic two-dimensional representations of the docking results were produced using LigPlot [52].

## Results and Discussion

### Helix prediction, multiple sequence alignment and homology modeling

**Fig. 1:**
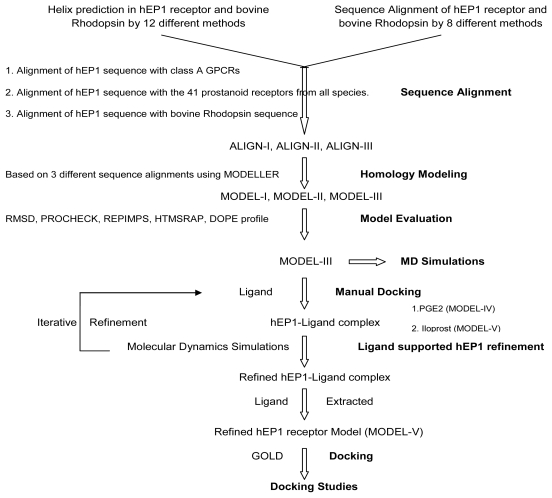
Flowchart of procedures for homology modeling of the hEP1R based on the known 3D structure of bovine rhodopsin as template.

GPCRs are major targets for drug development. The most common way to construct the structural model of their inactive/ground state is homology modeling using the crystal structure of bovine rhodopsin as template. Modeling quality is also correlated with sequence identity: the higher the similarity, the better the modeling, a fact which has been noted previously [53]. Human EP1R shares 18.6% and 32.6% of identity and similarity with visual rhodopsin respectively. The higher conservation seen in TM regions however is responsible for the similar overall folding of the members of GPCRs. Different strategies were used to carefully generate a set of feasible alignments between the sequences of our target protein and the template (Fig. 1).

**Fig. 2:**
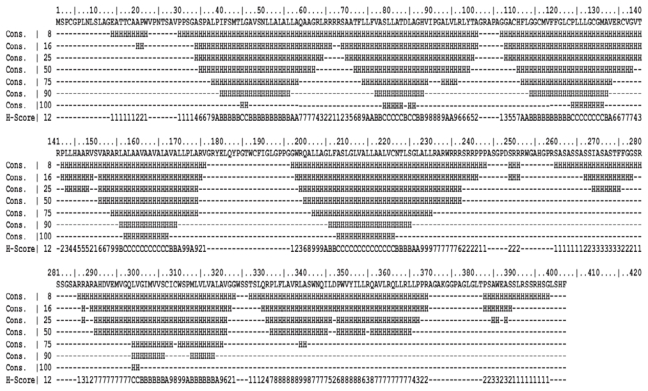
Consensus of 8, 16, 25, 50, 75, 90 and 100% of each residue in hEP1R predicted as helix by 12 different helix prediction methods. Each residue has a score from 1 to 12 based on how many methods predicted it as helix. A, B and C in scoring scale are equal to 10, 11 and 12. Consensus 50% comprises residues which have 6 or more scores.

The prediction of TM helices plays an important role in the study of membrane proteins, given the relatively small number (0.5% of the PDB) of high resolution structures available for such proteins. Here the prediction methods were assessed by bovine rhodopsin with known 3D structure and hEP1R. The distributions of helix lengths in the two proteins were examined (Table 2) and consensus 8%, 16%, 25%, 50%, 75%, 90% and 100% of methods which predicted α-helices for each sequences were assigned (Fig. 2). All of these methods work with different algorithm and predict different lengths and sizes of helices. As shown in Table 2 all methods predicted seven TM helices except TMHMM, MEMSAT and TopPred II which predicted 6 TM helices for hEP1R. Also TM regions of bovine rhodopsin predicted by using these methods were somehow different from DSSP result (data not shown). The 50% consensus for rhodopsin was almost compatible with DSSP assignment (data not shown) and hence was chosen for assessment of helices predicted in the constructed models.

**Fig. 3:**
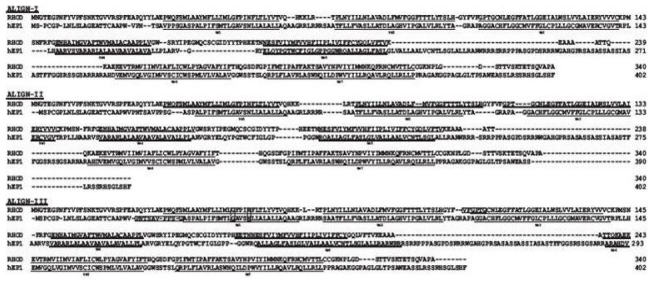
Alignment of hEP1R and bovine rhodopsin receptor by three different methods. ALIGN-I extracted from global alignment of all proteins belong to the rhodopsin-like family. ALIGN-II and III are between 41 prostanoid receptors from all species and hEP1R with bovine rhodopsin receptor, respectively (by ClustalW). ALIGN-III was modified manually in some regions (indicated by boxes) to protect conserved regions. Seven TMs are depicted by underlined letters.

The basic information from multiple alignments of the protein sequences are the position and nature of the conserved regions. All three alignments employed in the protocol have covered some of these conserved residues (Fig. 3). For ALIGN-III a manual adjustment was done to keep Gx(3)N conserved region (Table 1) in the right position and moving a gap from TM3 to EL1. Table 3 shows the alignment of hEP1R and bovine rhodopsin, identified in the GPCR database (ALIGN-I) was rather unique, particularly in TM5, and there was less similarity in TM regions between ALIGN-I and 50% consensus than the two other alignments. According to Table 3, ALIGN-II and III were more compatible with 50% consensus in the helix prediction.

Based on the sequence alignments, MODELLER extracts a large number of spatial restraints from the template structures and builds a molecular model of the query protein. The restraints are generally obtained by assuming that the corresponding distances and angles between aligned residues in the template and the target structures are similar. The resulting output was a homology model of hEP1R.

Three criteria were considered to distinguish the best model: RMSD of the transmembrane regions relative to rhodopsin, stereochemical quality of the models and REPIMPS scores as summarized in Table 4.

To examine the orientation of the helices of hEP1R, we superposed the transmembrane regions of the models with the template. From the RMSD values for hEP1R and rhodopsin, we can conclude there is a good agreement between the helices especially in MODEL-III.

Also, evaluation of the stereochemical quality of the models with PROCHECK showed that only a few residues are in disallowed regions in the Ramachandran plot, and most of them correspond to the structurally non-conserved regions. As it is evident from Table 4, all models have a very close and good stereochemical quality.

To assess the quality of the models, the residue environment compatibility scores were calculated for the generated hEP1R models using the REPIMPS method [40]. Also rotational orientation angles (α) for the TM helices were calculated for each of the 7 TM segments after idealizing them in terms of secondary structure. The results of the predictions for all the TM helices using HTMSRAP are summarized in Table 5. It shows that most of the helices, especially in MODEL-III, are acceptably faced to the lipid bilayer.

MODEL-III was chosen for further refinement steps because it had the highest lipid compatibility score, its helices were better oriented against each other and against hydrophobic area, and its RMS deviation relative to rhodopsin was the least one.

DOPE (Discrete Optimized Protein Energy) [54] which is implemented in MODELLER was used to assess the energy and the quality of the MODEL-III as a whole. The problematic regions in the MODEL-III were EL2, IL3 and the C-terminus, which were subjected to an *ab initio* loop modeling procedure implemented in MODELLER (data not shown).

Prostanoid receptors have several of the key residues and motifs characteristic of GPCRs. The extracellular domain of the hEP1R includes two NXS/T (Asn-X-Ser/Thr) consensus sequences for N-linked (Asn-linked) glycosylation. These motifs are located at Asn8 and Asn25 on the N-terminus. Investigations of the functional role of prostanoid receptor glycosylation revealed effects on ligand binding, receptor activation, and membrane localization [55].

A conserved disulfide bond, found in most GPCRs, is formed in the extracellular domain of the hEP1R between cysteine residues Cys110 (at the top of TM3) and Cys188 (EL2). A highly conserved (100% across prostanoid receptors) Arg338 is located in the middle of TM7, and thought to be analogous to Lys296 of rhodopsin, which is crucial for anchoring the ligand within the binding domain. Also located within TM7 of the hEP1R, and 100% conserved across all prostanoid receptors, is a DPWXY (Asp-Pro-Trp-X-Tyr) motif, which has been shown to be important for receptor activation.

### Molecular dynamics simulation and ligand-supported hEP1R model refinement

**Fig. 4:**
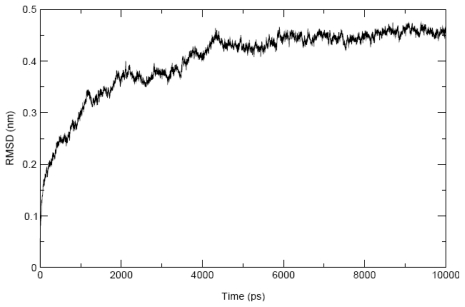
RMSD of alpha carbons vs. simulation time for 10 ns free molecular dynamics simulation on MODEL-III.

**Fig. 5:**
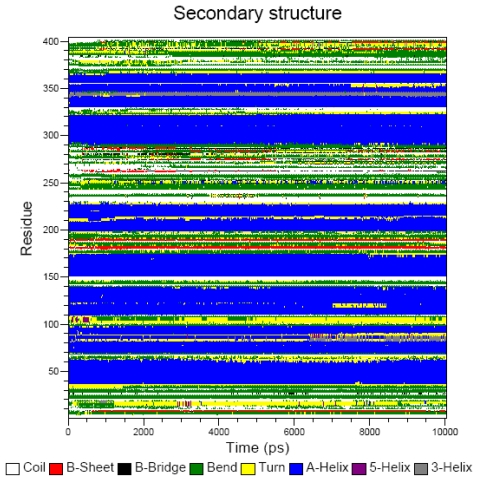
Evolution of the secondary structure of MODEL-III during 10 ns molecular dynamics simulation.

**Fig. 6:**
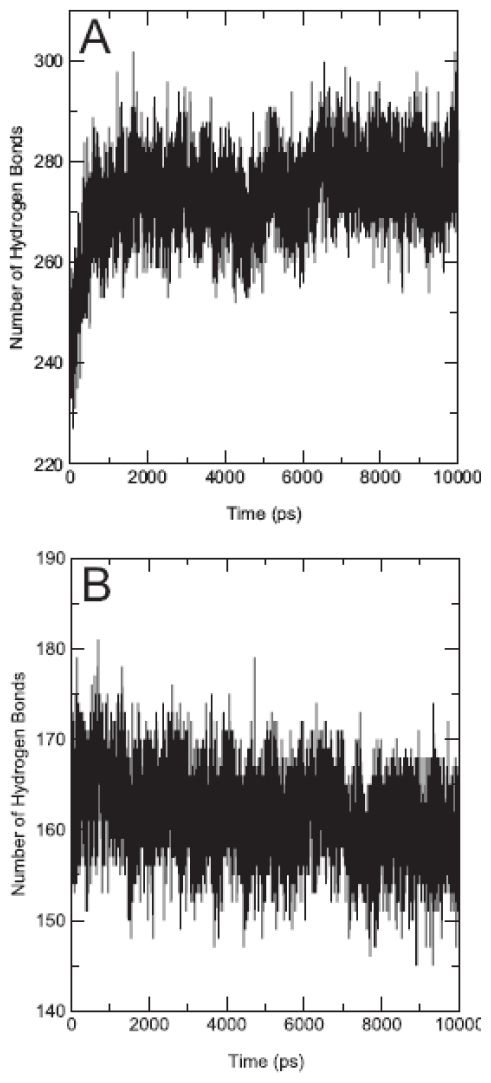
Number of hydrogen bonds during 10 ns MD simulation. (A) Number of all hydrogen bonds. (B) Number of hydrogen bonds for transmembrane helices.

During 10 ns unrestrained MD simulation of MDDEL-III, the total system energy (E_tot_) and the total energy of the protein (E_prot_) approached their minimum values after 6 ns and 8 ns, respectively. The seven TM helices remained within a Cα atom RMSD of 2.0 Å from the starting structure during this period. The maximum RMSD value for all Cα atoms was 4.5 Å (Fig. 4), indicating sufficient stability of the model. The protein appears to be equilibrated after 5 ns where RMSD approached the maximum. We also analyzed the evolution of the secondary structure of the hEP1R (Fig. 5). Overall, the seven TM segments conserved their α-helical secondary structure during the entire simulation. Fig. 6 shows change of the number of hydrogen bonds for the whole hEP1R structure and the transmembrane helices, respectively, during the simulation. As can be seen, the average number of hydrogen bonds increases from 240 to 275 (Fig. 6A) while the average number of hydrogen bonds in the seven transmembrane helices remains nearly constant (decrease from 165 to 159, Fig. 6B). Also, the rotational orientation angles measured by HTMSRAP before and after 10 ns simulation are almost unchanged (data not shown).

**Fig. 7:**
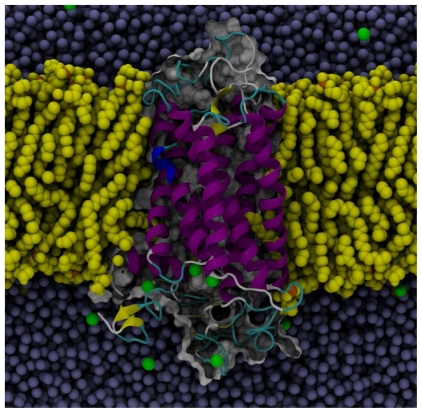
Molecular dynamics simulation box of hEP1R (MODEL-III) with lipid bilayer (yellow), chlorides (green) and waters (blue). Structure after 10 ns of simulation. The EC region is at the top. Phosphate atoms of lipids are shown as orange balls. The front half of the bilayer and two thirds of waters are not shown for the sake of clarity.

Together, these results indicate that the hEP1R structure was stable during the course of the MD simulation. Fig. 7 shows the structure of the hEP1R incorporated into lipid bilayer after 10 ns of simulation.

By analyzing the secondary structure (Fig. 5) and the hydrogen bonds of helices (Fig. 6) it can be seen that the structural changes during the MD simulation are very subtle. After 10 ns MD simulation, the seventh transmembrane helix was positioned in a better orientation with respect to the lipid bilayer (80%), while other helices nearly retained their orientations prior to the simulation.

There are three important interhelical hydrogen bonds in the structure that are all maintained during the 10 ns MD simulation. In TM1 there is a conserved G(x3)N motif that in bovine rhodopsin participates in a hydrogen bond. In our model this hydrogen bond between Gly46 and Asn50 exists and remains during 10 ns MD simulation. Also in the conserved DP(x2)Y motif of TM7 there is a hydrogen bond between Asp347 and Tyr351 which is maintained after 10 ns MD simulation. Arg338 in TM7, the key residue in the binding site, forms a hydrogen bond with Ser341 which is maintained during the 10 ns MD simulation.

All of these findings showed that the hEP1R structure was stable during the course of the MD simulation and is structurally feasible.

In most cases, homology models of GPCRs derived from bovine rhodopsin are not directly suitable for use in structure-based drug design and require targeted iterative refinement of the receptor binding site. It has been suggested that the binding sites of GPCR homology models are often too small to accommodate known ligands [50, 56]. The narrow binding pocket is possibly the result of the flat nature of 11-cis retinal in the binding pocket of the bovine rhodopsin crystal structure and the misplacement of side-chains of binding site residues during homology modeling [50].

To address this problem, a balloon potential involving a systematic MD-based methodology to expand and refine the binding sites of models has been reported and applied successfully to several GPCR receptors [50, 56]. In the current work, the hEP1R model (MODEL-III) was subjected to the similar ligand-supported homology model refinement protocols described previously [50].

**Fig. 8:**
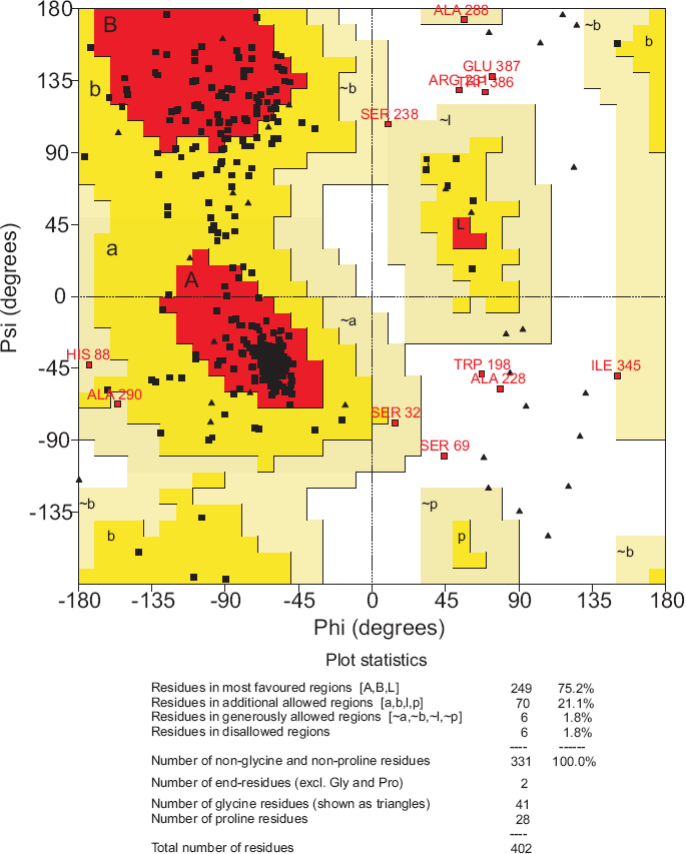
Ramachandran plot analysis of the final hEP1R model (MODEL-V).

It was performed in two steps. First the refinement was employed with PGE_2_, and then the resulting model (MODEL-IV) was subjected to another refinement processes with iloprost docked to the binding pocket. After calculating the average structure achieved in the final MD simulation this structure was subjected to a series of minimization steps (MODEL-V). MODEL-V can now explain ligand–receptor interactions for some prostanoid compounds. Appropriate care was taken throughout the refinement protocol to maintain the conserved α-helical geometry of the TM helices. Evaluation of the stereochemical quality of MODEL-V with PROCHECK showed that only a few residues are in disallowed regions in the Ramachandran plot and all of them correspond to the structurally non-conserved regions (Fig. 8). The six non-glycine residues, Trp198, Ala228, Ser69, Arg280, Asp387 and Trp386 were observed lying outside the allowed regions. These residues in the extracellular and intracellular loop regions do not belong to the binding site.

**Fig. 9:**
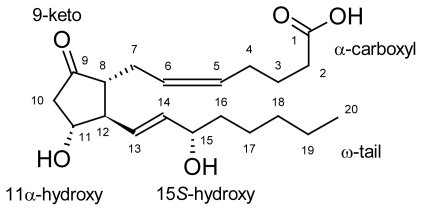
Structure of PGE_2_. Carbon atoms are numbered from 1 to 20 starting from the C-1 carboxyl group.

In order to investigate the validity as well as the characteristics of the binding site of our model and to facilitate the rational design of novel and more selective prostanoid receptor ligands, docking analysis was performed with PGE_2_, a potent analogue of hEP1R and some other prostanoid ligands. PGs have two structural features, a cyclopentane ring and two side chains, and the receptors are supposed to recognize all these moieties and stabilize the ligand binding (Fig. 9).

**Fig. 10:**
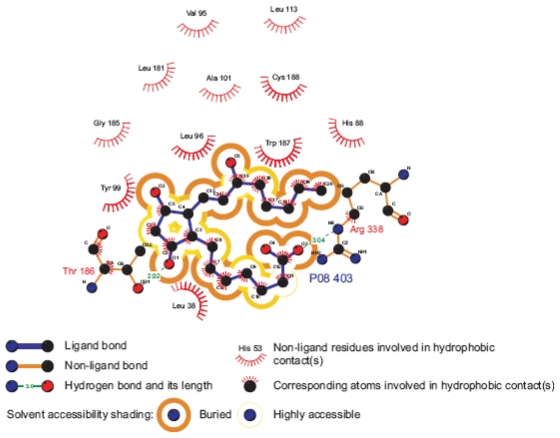
Schematic two-dimensional representations of the binding interactions between PGE_2_ and hEP1R

Docking analysis revealed that the putative binding domain of the prostanoid receptors lie within the upper half of the transmembrane-spanning region. This pocket is formed mainly by the first, second and seventh transmembrane segments, of which the former two are involved in the recognition of the ring structure and the latter in that of the side chains (Fig. 10). Also EL2 covers the binding site and could construct a hydrogen bond with the cyclopentane ring of the ligand via Thr186. The key interactions of PGE_2_ with the hEP1R have been highlighted by site-direct mutagenesis experiments as follows:

Arg338 in TM7 is supposed to form a salt bridge with the carboxylic acid moiety on the α chain of PGE_2_. Strong evidence in support of this interaction is that the mutation of the corresponding Arg in prostaglandin I [57], EP2 [58], EP3 [59], prostaglandin F [60], and thromboxane [61] receptors leads to significant loss of binding. Such mutation experiments have not yet been carried out for the hEP1R. However, as Arg338 is conserved in all prostanoid receptors, the proposed interaction between 1-COOH of PGE_2_ and Arg338 is obvious. Mutation of Tyr99 into Ala in the human prostacyclin receptor, corresponding to Tyr75 (TM2) of hEP1R, leads to a significant change of the binding affinity of iloprost [55]. It has been suggested that Tyr75 interacts with 11-OH of the cyclopentane ring of hIP. Tyr99 may participate in hydrogen binding with 9-OH of the cyclopentane moiety and hydrophobic interaction with the ring. Some mutation studies also revealed the role of EL2 on hydrogen bonding with the ligand and the participation of TMs 1-3 in hydrophobic interactions with the α and ω chains of PGE_2_ in hEP3R and IPR [55, 62].

In our model, the binding site has a hydrophobic pocket consisting of several residues. This hydrophobic pocket surrounds the α and ω chains and is involved in PGE_2_ binding. In some studies substitution of His81 of rat prostaglandin F and thromboxane receptors, corresponding to His88 in hEP1R, with a number of different amino acids, led either to a loss of ligand binding or to alterations in the optimum pH for ligand-receptor interaction [63]. Therefore, these interactions are likely important for PGE_2_ binding with hEP1R.

Table 6 shows a comparison of the receptor affinity, obtained previously by Ungrin et al. [48], with the docking score, using GOLDscore, for all compounds docked to the hEP1R.

**Fig. 11:**
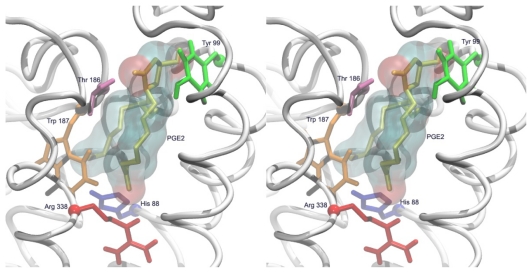
Stereoview of the predicted binding site of PGE_2_ in MODEL-V. Five important residues in the pocket are indicated by different colors.

Ungrin et al. described SAR of prostanoids and prostanoid analogues at the hEP1R [48]. They obtained experimental binding affinities for PGE_2_ and related compounds. The data revealed that 11α and 15(S) configuration of the hydroxyl groups as well as the presence of the carboxylic acid moiety of the prostanoid structure are crucial for hEP1R binding and activation. Here we used 15 prostanoid compounds which have covered some structural changes to achieve different binding affinity. Each of these compounds was docked into the binding site of the hEP1R for 100 runs. The structures were scored with GOLDscore, and correlation analysis of receptor binding affinities (Ki) and docking scores was performed for evaluation of the ligand-receptor complexes modeled in this study. Compounds in the first half of Table 6 are potent agonists with sub-micromolar Ki values, while the others are weak agonists. A squared correlation coefficient of r^2^=0.81 between the Ki values and the GOLDscores was observed. The docking study was performed on the homology-derived MODEL-V, and for such a study this correlation is quite reasonable and indicates an acceptable quality of the model. Comparison of the best docking modes of various compounds suggests common binding characteristics very similar to that depicted for PGE_2_ in Fig. 11.

## Conclusions

We have provided a homology model of the human prostanoid receptor, hEP1R, based on the structure of bovine rhodopsin (1L9H). Models of ligand–protein complexes have been derived by docking studies. The predicted binding site was in the cavity inside the receptor between TMs 1,2,3 and 7 and the EL2 region of the hEP1R. Arg338 in TM7 is a key residue for ligand binding and receptor activation. Thr186 in EL2 as well has an important role. Hydrogen bonds and hydrophobic interactions with other residues further contribute to ligand binding. The identified key residues involved in ligand-receptor interactions are in agreement with results from in vitro mutagenesis. Since the prostanoid receptors are interesting therapeutic targets, the results of the current study provide information which can be used in structure-based drug design and may initiate further biological experiments.

## Figures and Tables

**Tab. 1:** Key residues conserved in the GPCR family.

TM	Pattern	Bovine rhodopsin	hEP1
TM1	Gx^a^(3)N	+	+
TM2	Lx(3)Dx(7)P	Lx(3)D	Lx(3)D
TM3	Sx(3)Lx(2)Ix(2)DRY	+	Px(3)Gx(2)Mx(2)ERC
TM4	Wx(7,8)P	+	Ax(9)P
TM5	Fx(2)Px(7)Y	+	P
TM6	Fx(2)CWxP	+	SX(2)CWxP
TM7	Lx(5)NPx(2)Y	Kx(5)YNPx(2)Y	Lx(7)DPx(2)Y

a Any residue.

**Tab. 2:** Locations of helical TM segments of hEP1R predicted by 12 different commonly used methods.

Helix	PHD	PROF	JPRED	SAM–T99	Porter	PSIPRED	Bovine rhodopsin Crystal structure (DSSP)
I	39–56	34–46	45–66	30–62	38–66	34–63	34–64
II	79–96	75–88	77–86	72–101	69–100	70–100	71–100
III	114–131	111–138	124–138	99–140	99–138	98–139	106–139
IV	157–174	153–167	147–170	144–174	146–173	151–174	150–172
V	205–227	202–237	200–237	199–243	197–237	200–236	196–223
VI	299–318	290–306	290–300	287–322	287–321	290–321	243–277
VII	339–356	332–340	331–341	330–369	327–365	332–367	285–321

Helix	SABLE2.0	SCRATCH	HMMTOP	TMHMM	MEMSAT	TopPred II
I	35–62	35–64	34–58	40–62	37–58	38–58
II	73–100	71–101	71–95	74–96	73–96	68–88
III	97–140	98–140	112–133	111–133	112–140	112–139
IV	151–175	151–174	156–174	154–176	156–174	154–174
V	200–237	198–241	201–225	208–230	206–230	201–221
VI	286–320	290–321	299–320	299–321	299–323	299–319
VII	332–365	331–369	333–354	—	—	—

**Tab. 3:** TM segments predicted by different alignment strategies compared to TM segments identified by 50% consensus of TM prediction.

TM	50% consensus	ALIGN–I^a^	ALIGN–II^b^	ALIGN–III^c^
TM1	35–62	29–58	37–59	34–62
TM2	72–99	72–100	72–98	69–98
TM3	111–138	107–140	108–139	111–139
TM4	151–174	146–169	151–173	151–173
TM5	200–237	183–209	199–227	197–224
TM6	290–321	292–321	290–321	287–321
TM7	332–354	330–354	330–354	330–354

a Rhodopsin–like GPCR family;

b Prostanoid receptors/rhodopsin;

c hEP1R/rhodopsin.

**Tab. 4:** Comparison of the model fitness criteria among the three models.

Model	RMSD of the TM Cα from rhodopsin (Å)	Procheck analysis		Lipid compatibility score (REPIMP)

		Allowed regions	Disallowed regions	
MODEL–I^a^	0.95	99.0%	0.9%	121.37
MODEL–II^b^	0.88	99.0%	0.9%	135.32
MODEL–III^c^	0.63	98.8%	1.2%	140.50

a Based on the rhodopsin-like GPCR alignment;

b Based on prostanoid receptors/rhodopsin aligment;

c Based on hEP1/rhod alignment.

**Tab. 5:** Rotational orientation angles (α) of transmembrane helices for three different models of hEP1R predicted using HTMSRAP method based on the π propensity scale, and percentages of the accessible surface area of the helices exposed to the lipid bilayer.

	MODEL-I		MODEL-II		MODEL-III	
	
	α angle a	% b	α angle a	% b	α angle a	% b
TM1	139.19	100	243.02	40	18.91	60
TM2	17.1	45	33.64	30	327.2	85
TM3	49.17	30	204.8	50	47.45	60
TM4	32.28	60	302.7	20	302.7	20
TM5	218.68	30	295.11	80	295.11	80
TM6	302.26	71	302.26	71	146.03	85
TM7	104.82	71	104.82	71	104.82	71

a Based on π scale on HTMSRAP;

b Percent of amino acids on the lipid side of each helix compared with the ideal helix

**Tab. 6:** Docking scores and Ki values for prostanoid compounds.

Compound	Ki^a^ (μM)	Docking Score^b^
9-Deoxy-9-methylene-prostaglandin E2	0.007425	64.9779
17-Phenyl-v-trinor-prostaglandin E2	0.004257	61.8478
17-Phenyl-v-trinor-prostaglandin F2a	0.05346	62.2498
Prostaglandin E3	0.0792	61.346
Prostaglandin F2a	0.3762	62.3059
15-Cyclohexyl-pentanorprostaglandin F2a	0.8613	63.3909
Latanoprost free acid	1.188	64.6217
11-Deoxy-prostaglandin E2	1.188	64.0851
8-iso-Prostaglandin E2	1.188	61.6362
15-keto-Prostaglandin E2	2.376	62.1678
Misoprostol free acid	14.85	54.8817
15(R)-Prostaglandin E1	16.83	53.8978
8-iso-Prostaglandin F2a	8.91	59.8972
Prostaglandin E2 ethanolamide	9.603	58.7369
Misoprostol methyl ester	46.53	50.5039

a Experimental Ki values by Ungrin et al. [48],

b The structures were docked with GOLD and scored by GOLDscore.
